# Facilitators and barriers for working beyond statutory pension age: A prospective cohort study across 26 European countries

**DOI:** 10.5271/sjweh.4189

**Published:** 2024-12-01

**Authors:** Lars Louis Andersen, Joaquín Calatayud, Rodrigo Núñez-Cortés, Ana Polo-López, Rubén López-Bueno

**Affiliations:** 1 National Research Centre for the Working Environment, Copenhagen, Denmark.; 2 Exercise Intervention for Health Research Group (EXINH-RG), Department of Physiotherapy, University of Valencia, Spain.; 3 Department of Physical Therapy, Faculty of Medicine, University of Chile, Santiago, Chile.; 4 Department of Physical Medicine and Nursing, University of Zaragoza, Zaragoza, Spain.

**Keywords:** influence, older worker, prospective cohort study, psychosocial, retirement, skill development, statutory retirement age, time pressure, work factor

## Abstract

**Objective:**

The aging population of European countries highlights the need for extended working lives. This study aims to investigate facilitators and barriers for working beyond the statutory pension age (SPA).

**Methods:**

Using data from waves 1, 2, 4–9 of the Survey of Health, Ageing and Retirement in Europe (SHARE) (2004–2022), we followed 9131 workers with a mean age of 56.9 [standard deviation (SD) 3.5] years from 26 European countries until they surpassed the SPA for their respective country, sex and year of participation. Using robust Poisson regression, we modelled the prospective association of work factors, lifestyle, health, and demographics at baseline with working at least one year beyond the SPA.

**Results:**

Participants were followed for 9.5 (SD 3.9) years. After surpassing the SPA by at least one year, 18% were still working. Among the work factors, opportunities for skill development [risk ratio (RR) 1.20, 95% confidence interval (CI) 1.07–1.34] and recognition at work (RR 1.13, 95% CI 1.01–1.26) facilitated working beyond SPA, while time pressure (RR 0.89, 95% CI 0.81–0.97) and poor prospects for job advancement (RR 0.76, 95% CI 0.70–0.83) were barriers. For the other factors, smoking was negatively associated with working beyond the SPA, while living in the northern part of Europe, higher level of education, and being divorced or separated were positively associated with working beyond the SPA.

**Conclusion:**

This prospective cohort study across 26 European countries identified four modifiable work factors that influenced working beyond the SPA. Addressing modifiable barriers and facilitators at the workplace and through public health initiatives could help extend working lives in Europe.

As life expectancy continues to increase in Europe, the proportion of older individuals is growing rapidly, leading to a steady increase of the old-age dependency ratio. This challenges the sustainability of social security systems and the overall economy of countries in the European Union (1). To counteract this, many European countries have implemented policies to raise the statutory pension age (SPA) and discourage early retirement (2). For example, Denmark has raised the SPA from 65 to 67 years in the period from 2018 to 2022. However, extending working lives should go beyond merely raising the SPA. It should also involve promoting workplace and societal factors that encourage people to continue working to a higher age because they find it fulfilling and beneficial. Analyzing factors associated with working beyond the SPA provides a unique opportunity to identify facilitators and barriers influencing individuals’ decisions to continue working, even when they have the opportunity to retire and receive pension benefits.

Systematic reviews have found work engagement and burnout, respectively, are positively and negatively associated with working beyond the normal retirement age, showing that both motivation and capability are important factors (3, 4). The Dutch Study on Transitions in Employment, Ability and Motivation showed that health, workload, and the individual’s financial situation were important factors for working beyond retirement (5, 6). A Finnish study found that managers and professionals were more likely to work beyond retirement compared to workers in skilled manual and elementary occupations, largely due to having lower workload, better work time control, and higher work ability (7). A Danish study showed that a good psychosocial work environment facilitated working beyond retirement age, both among workers with seated and physically active work (8). Altogether, these studies point towards several modifiable work factors for promoting longer and healthier working lives.

While previous research in this area have provided valuable knowledge, most studies have been limited to single countries or used cross-sectional designs. Thus, prospective cohort studies spanning various countries are necessary to provide broader knowledge on facilitators and barriers for working beyond SPA in Europe. Such an approach is essential for identifying modifiable factors that can be targeted through interventions at the workplace and public health initiatives to promote extended working lives. In addition, previous studies examined the likelihood of labor market exit at a predetermined age (eg, 65 years) across all participants. However, this approach does not account for the fact that the SPA varies between women and men in several European countries, as well as across different years and countries (2, 9, 10). Our study addresses this limitation by using country-, sex-, and year-specific SPA as reference points.

The primary aim of this study was to investigate work factors that may act as facilitators or barriers for working beyond the SPA in Europe, such as time pressure at work, skill development opportunities, and work recognition. As a secondary focus, we also examine the potential influence of sociodemographic factors (eg, education level, marital status), lifestyle factors (eg, smoking status), and health status. We used data from the Survey of Health, Ageing and Retirement in Europe (SHARE) study, a large multinational cohort of individuals aged ≥50 years, combined with country-, sex- and year-specific SPA.

## Method

### Study design and participants

This study uses data from eight waves (1, 2, 4–9) of the SHARE study, which includes 28 countries (27 European countries and Israel) (11, 12). Not all individuals participated in the same waves and, to obtain a larger sample size, we therefore utilized all waves. Data collection took place from February 2004 to December 2022. We excluded wave 3 as it did not include the variables used for the present analyses. We did not include Israel as we focused on European pension systems.

SHARE employs a multistage stratified sampling design. In this design, participating countries are divided into different strata based on their geographical area, with municipalities or zip codes within these strata serving as primary sampling units (11). SHARE collects data every two years through home computer-assisted personal interviews, using ex-ante harmonized questionnaires. To compensate for attrition, new respondents are added in each wave.

The target population for SHARE consists of all individuals aged ≥50 years at the time of sampling who have their regular domicile in the respective SHARE country. Individuals are excluded from the baseline or refreshment samples if they are incarcerated, hospitalized, or out of the country during the entire survey period, unable to speak the country’s language(s), or have moved to an unknown address. Partners living in the same household as the target population are interviewed regardless of their age.

**Figure 1:**
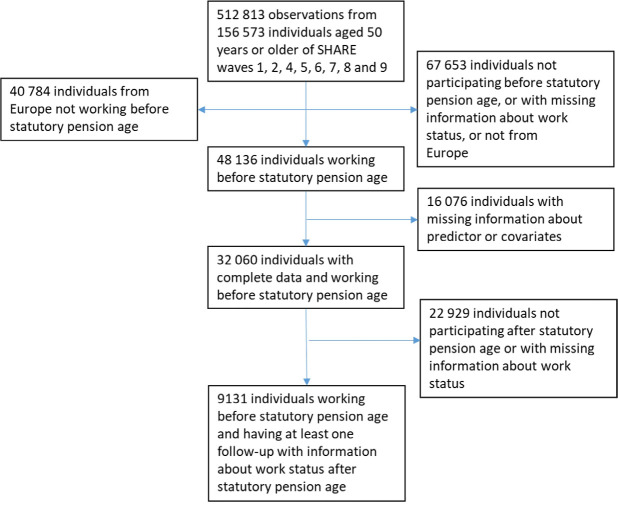
Flow of participants through the study.

The combined eight waves (1, 2, 4–9) contained 512 813 observations from 156 573 individuals aged ≥50 years. Among these, 48 136 individuals worked before the SPA and 32 060 of these had complete baseline data, of which 9131 had a follow-up at least one year beyond the SPA with information about work status (working or not working). Baseline was defined as the most recent interview conducted prior to the participant reaching the SPA, while they were still working. Thus, the final sample size consisted of 9131 individuals. None of the participants from Latvia fulfilled the inclusion criteria, and the total number of countries in the final sample was therefore 26. Figure 1 shows the flow of participants through the study.

### Statutory pension age

The SPA is the minimum age set by law at which an individual can begin receiving state pension benefits. As the SPA is specific to each country, sex and year, we retrieved historical information about this through three sources: (i) social security programs throughout the world (9), (ii) the OECD Data Explorer (10), and (iii) the International Social Security Association (2). The supplementary material (www.sjweh.fi/article/4189), figures S1–4, shows the development of SPA for men and women, respectively, across the 26 European countries and the four European regions (2004–2022). This information was used to define the respondents’ SPA specific to each country, sex and year. We did not consider the varying possibilities for early retirement benefits in the different countries as these are dependent on many other criteria than age. Furthermore, we were interested in analyzing working beyond the SPA and not early retirement. We defined “beyond the SPA” as at least one year beyond the SPA specific to each country, sex and year.

### Explanatory factors and covariates

We included work factors, lifestyle, health, and demographics at baseline as explanatory factors. For the work factors, we included the following nine statements: (i) my job is physically demanding; (ii) I am under constant time pressure due to a heavy workload; (iii) I have very little freedom to decide how I do my work; (iv) I have an opportunity to develop new skills; (v) I receive adequate support in difficult situations; (vi) I receive the recognition I deserve for my work; (vii) considering all my efforts and achievements, my [salary is/earnings are] adequate; (viii) my [job promotion prospects/prospects for job advancement] are poor; and (ix) my job security is poor. These nine items were weakly correlated. The highest Spearman’s r value was 0.44 between item 5 and 6. For all questions, the response scale was strongly agree, agree, disagree, and strongly disagree. For subsequent analyses, we dichotomized this into agree (ie, strongly agree and agree) and disagree (ie, disagree and strongly disagree). Furthermore, we constructed an “overall work factors” score as the average value of the nine questions normalized on a continuous scale (0–1), where agreeing with the positively phrased statements (iv–vii) each equaled 1 and agreeing with the negatively phrased ones (i–iii, viii, ix) each equaled 0, and vice versa. This allowed us to make a direct comparison of the risk estimates from the overall score with the nine individual work factors.

For the other factors, we considered the following explanatory factors: sex, smoking, body mass index (BMI), marital status, education, geographical region, and self-rated health. The interviewer noted the sex of the respondent based on observation and asked the respondent in case of uncertainty. Smoking status was based on two questions about whether the respondent had ever smoked and was currently smoking, and was categorized into one variable (current smoker, ex-smoker, never smoked). BMI was calculated from self-reported weight and height of the respondent and categorized into <18.5, 18.5–<25, 25–<30, and ≥30 kg/m^2^. Respondents also replied to a question with six categories about marital status that was combined into four categories (i) married and living together with spouse or in a registered partnership, (ii) divorced or married, but living separated from spouse, (iii) never married, or (iv) widowed. Education was based on the question “What is the highest school leaving certificate or school degree that you have obtained?” with response categories recoded into three ISCED-1997 categories (i) lower (pre-primary education, primary education or first stage of basic education, or lower secondary or second stage of basic education); (ii) medium (upper secondary education, or post-secondary non-tertiary education); and (iii) higher education (first stage of tertiary education, or second stage of tertiary education) (13). Country (26 categories: Austria, Belgium, Bulgaria, Croatia, Cyprus, Czech Republic, Denmark, Estonia, Finland, France, Germany, Greece, Hungary, Italy, Lithuania, Luxembourg, Malta, Netherlands, Poland, Portugal, Romania, Slovakia, Slovenia, Spain, Sweden, Switzerland) was recoded into a new variable, geographical region, with four categories (i) Eastern, (ii) Western, (iii) Southern and (iv) Northern Europe, according to the United Nations definition (United Nations 2024). Self-rated health was based on the question “Would you say your health is...” with the five response options excellent, very good, good, fair and poor, which was categorized into (i) excellent, (ii) good to very good and (iii) poor to fair health.

Furthermore, we included age at the time of the interview, calculated as date of interview minus date of birth, and time beyond the SPA, defined as age at the follow-up interview minus the SPA specific to country, sex and year. We included these two covariates in our statistical analyses to account for variations in the SPA across countries and over time, as well as differences in time beyond the SPA when the follow-up interview was performed, which may influence the probability of working.

### Outcome: work status

Work status was defined through the question “In general, which of the following best describes your current employment situation?” with the response options retired; employed or self-employed (including working for family business); unemployed and looking for work; permanently sick or disabled; homemaker; and other (rentier, living off own property, student, doing voluntary work). Only participants who were employed or self-employed at baseline were included in the sample at baseline. At follow-up, the same question was used determine work status (working or not working) as the outcome variable.

### Statistical analyses

Using the generalized linear models (GENMOD) procedure of SAS version 9.4 (SAS Institute, Cary, NC, USA), we conducted robust Poisson regression analysis (14) to assess the prospective association between the aforementioned explanatory factors at baseline and work status at follow-up. We chose robust Poisson regression over logistic regression because the outcome of working beyond SPA was common (>10% prevalence). We performed two separate multivariable analyses.

The first analysis included all nine individual work factors simultaneously, along with all other explanatory factors (demographics, lifestyle, and health) and covariates (age at baseline and time since SPA). This allowed us to examine the independent association of each work factor with working beyond the SPA while controlling for all other variables in the study.

The second analysis replaced the nine individual work factors with the overall work environment score (continuous score from 0–1), while still including all other explanatory factors and covariates. This allowed us to examine the association of overall work environment with working beyond the SPA while controlling for all other variables in the study.

Additionally, we performed two sensitivity analyses, where we included occupation (Model 2) and excluded self-rated health (Model 3), respectively.

Results are reported as risk ratios (RR) with 95% confidence intervals (CI). RR>1 means that there is an increased chance of working beyond the SPA (ie, facilitator), while RR<1 means that there is a decreased chance of working beyond SPA (ie, barrier). We considered results statistically significant at P<0.05.

## Results

Table 1 shows the baseline characteristics of the study population. The mean age at baseline was 56.9 (SD 3.5) years. The proportion of men (52%) and women (48%) was quite alike. The majority of participants had a BMI of 18.5–30 kg/m^2^ (82%), and were married and living with their spouse or in a registered partnership (77%). The proportion of participants who had never smoked was 46%. The majority reported good-to-excellent health (82%). The largest proportion resided in Western Europe (43%).

**Table 1:** Descriptive baseline characteristics of the study population. [SD=standard deviation; freq=frequency.]

		N	Mean	SD	Freq (%)
Age		9131	56.9	3.5	
Sex					
	Men	4788			52
	Women	4343			48
Smoking status					
	Never smoked	4238			46
	Ex-smoker	2732			30
	Current smoker	2161			24
Body Mass Index (BMI)					
	<18.5	67			1
	18.5–<25	3640			40
	25–<30	3851			42
	≥30	1573			17
Education (ISCED-1997)					
	Lower	2348			26
	Medium	3784			41
	Higher	2999			33
Marital status					
	Married and living together with spouse or in a registered partnership	7053			77
	Divorced or married but living separated from spouse	1135			12
	Never married	585			6
	Widowed	358			4
Self-rated health					
	Excellent	1393			15
	Good or very good	6112			67
	Poor or fair	1626			18
Region of Europe (United Nations definition)					
	Eastern	1005			11
	Northern	2554			28
	Southern	1669			18
	Western	3903			43

The mean follow-up time of the 9131 participants was 9.5 (SD 3.9) years and the mean age at follow-up was 66.4 (SD 2.4) years. After surpassing the SPA by least one year, 1647 (18%) still performed paid work.

Table 2 presents the main analyses, ie, RR with 95% CI for working beyond the SPA associated with nine different work factors and the overall work factors score. The main analysis (Model 1) showed that opportunities for skill development at work was associated with 20% higher probability (ie, facilitator) of working beyond the SPA (RR 1.20, 95% CI 1.07–1.34). Similarly, recognition at work was associated with 13% higher probability of working beyond the SPA (RR 1.13, 95% CI 1.01–1.26). In contrast, poor prospects for job advancement were associated with 24% lower probability (ie, barrier) for working beyond the SPA (RR 0.76, 95% CI 0.70–0.83). Likewise, time pressure at work was associated with 11% lower probability of working beyond the SPA (RR 0.89, 95% CI 0.81–0.97). The overall work factor score, analyzed in a separate model, showed that scoring 1 versus 0 (ie, the extreme end of the continuous scale, corresponding to positive versus negative responses to all nine factors) was associated with 68% higher probability of working beyond the pension age (RR 1.68, 95% CI 1.35–2.07). Table 2 further shows that sensitivity analyses including occupation (Model 2) and excluding self-rated health (Model 3) did not change the overall results.

**Table 2:** Risk ratios (RR) with 95% confidence intervals (CI) for working beyond pension age from the nine different work factors as well as from the overall work environment score. The first column ‘%’ refers to the percentage agreeing and disagreeing with the statement, while the second column ‘Crude %’ refers to the percentage working beyond SPA in each of these respective categories.

			Working beyond statutory pension age					
Work factors			Crude %	Model 1 ^a^		Model 2 ^b^		Model 3 ^c^
		%		RR (95% CI)		RR (95% CI)		RR (95% CI)
Physically demanding								
	Agree	46	17	1.03 (0.94–1.13)		1.01 (0.92–1.11)		1.04 (0.95–1.14)
	Disagree	54	19	1		1		1
Time pressure								
	Agree	49	16	0.89 (0.81–0.97)		0.89 (0.82–0.98)		0.88 (0.81–0.96)
	Disagree	51	20	1		1		1
Lack of influence								
	Agree	26	15	0.98 (0.88–1.10)		0.97 (0.87–1.09)		0.99 (0.88–1.10)
	Disagree	74	19	1		1		1
Opportunities for skill development								
	Agree	71	20	1.20 (1.07–1.34)		1.22 (1.09–1.37)		1.19 (1.06–1.33)
	Disagree	29	13	1		1		1
Support in difficult situations								
	Agree	73	19	0.94 (0.84–1.05)		0.94 (0.84–1.05)		0.94 (0.84–1.05)
	Disagree	27	16	1		1		1
Recognition for work								
	Agree	71	19	1.13 (1.01–1.26)		1.12 (1.00–1.25)		1.13 (1.01–1.26)
	Disagree	29	15	1		1		1
Adequate salary								
	Agree	56	18	0.97 (0.89–1.07)		0.97 (0.89–1.07)		0.97 (0.88–1.06)
	Disagree	44	18	1		1		1
Poor prospects for job advancement								
	Agree	67	17	0.76 (0.70–0.83)		0.76 (0.69–0.83)		0.76 (0.70–0.83)
	Disagree	33	21	1		1		1
Poor job security								
	Agree	23	18	1.07 (0.96–1.18)		1.06 (0.96–1.18)		1.08 (0.97–1.20)
	Disagree	77	18	1		1		1
	Overall work factors (score 0–1) ^d^		N/A	1.68 (1.35–2.07)		1.76 (1.41–2.20)		1.61 (1.31–1.99)

^a^ Main analysis. The nine different work factors and the overall work environment score are mutually adjusted for each other, and for all the other predictors from Table 3, as well as age at the baseline interview and time span from the statutory pension age to the follow-up interview. ^b^ Sensitivity analysis, including the same variables as Model 1 and additionally occupation (ISCO 1-4 and 5-9). ^c^ Sensitivity analysis, including the same variables as Model 1 except self-rated health. ^d^ Overall work factors was analyzed in a separate model than the nine work factors: Mean 0.62 (SD 0.22.)

Table 3 shows the secondary analyses, ie, the risk ratios with 95% confidence intervals for working beyond the SPA associated with demographics, lifestyle, and health factors. Compared to those who never had smoked, smokers and ex-smokers had a 15% (RR 0.85, 95% CI 0.76–0.95) and a 13% (RR 0.87, 95% CI 0.79–0.96) lower probability of working beyond SPA, respectively. Participants with a medium and higher education level had a 43% (RR 1.43, 95% CI 1.26–1.63) and a 60% (RR 1.60, 95% CI 1.40–1.84) higher probability of working beyond the SPA compared to those with lower education. Divorced or separated participants had a 42% higher probability (RR 1.42, 95% CI 1.27–1.59) of working beyond SPA compared to those being married and living together. Eastern, Southern, and Western Europeans had a 55% (RR 0.45, 95% CI 0.37–0.54), 54% (RR 0.46, 95% CI 0.39–0.53), and 56% (RR 0.44, 95% CI 0.40–0.49) lower probability of working beyond the SPA, respectively, compared to Northern Europeans.

**Table 3:** Risk ratios (RR) with 95% confidence intervals (CI) for working beyond pension age from demographics, lifestyle and health.* The column ‘Crude %’ refers to the percentage working beyond SPA in each category.

Demographics, lifestyle and health		Working beyond statutory pension age	
		Crude %	RR (95%CI)
Sex			
	Man	20	1
	Woman	21	0.98 (0.90–1.08)
Smoking status			
	Never smoked	21	1
	Ex-smoker	20	0.87 (0.79–0.96)
	Current smoker	18	0.85 (0.76–0.95)
Body mass index (kg/m^2^)			
	<18.5	13	0.70 (0.36–1.36)
	18.5–<25	21	1
	25–<30	19	0.89 (0.81–0.98)
	≥30	21	0.99 (0.88–1.12)
Education			
	Lower	13	
	Medium	21	1.43 (1.26–1.63)
	Higher	25	1.60 (1.40–1.84)
Marital status			
	Married and living together with spouse or Registered partnership	19	1
	Divorced or married, but living separated from spouse	28	1.42 (1.27–1.59)
	Never married	20	1.16 (0.98–1.38)
	Widowed	24	1.22 (1.00–1.49)
Self-rated health			
	Good to excellent health	19	0.94 (0.83–1.05)
	Poor to fair health	24	1.10 (0.96–1.26)
Region of Europe			
	Northern	36	1
	Eastern	14	0.45 (0.37–0.54)
	Southern	14	0.46 (0.39–0.53)
	Western	15	0.44 (0.40–0.49)

* All explanatory factors in the model are mutually adjusted and are also adjusted for the nine different work factors from Table 2 (Model 1), as well as age at the baseline interview and time span from the statutory pension age to the follow-up interview.

## Discussion

Given the demographic shift towards an aging population across Europe, finding ways to extend working lives will be increasingly important for maintaining economic stability and supporting the well-being of older individuals who wish to continue working. This prospective cohort study among adults aged ≥50 years across 26 European countries identified several modifiable work factors that influence working beyond the SPA. Opportunities for skill development and recognition at work were facilitators, while time pressure and poor prospects for job advancement were barriers.

In line with a previous prospective cohort study from Denmark (8), the present findings show that opportunities for skill development facilitates working beyond the SPA. This highlights the importance of providing ongoing learning and growth opportunities for older workers. This also aligns with research showing that work engagement is an important factor in prolonging working lives (3, 4). However, it also points to potential disparities in the workforce, as opportunities for skills development may not be equally distributed across all job types or worker demographics. From a practical point of view, workplaces may consider investing in training and development programs to ensure a sense of purpose and motivation, in addition to ensuring adequate skills to keep up with changes, thereby encouraging older workers to continue working past typical retirement ages.

Recognition at work also facilitated working beyond the pension age. This suggests that acknowledging the contributions and expertise of older workers is important for maintaining engagement and commitment to the work. These findings align with previous studies showing that perceived organizational support and a positive work climate are associated with delayed retirement intentions (15, 16). Thus, workplaces should strive towards a culture that values and appreciates the experience and skills of older workers as this may contribute to overall job satisfaction and encourage extended working lives.

On the other hand, time pressure and poor prospects for job advancement were identified as barriers to working beyond the SPA. Regarding time pressure, this underscores the importance of psychological workload management and – if possible – providing opportunities for career growth to retain older workers. A previous systematic review of cross-sectional and longitudinal studies found mixed results from high job demands (eg, experienced as time pressure) on early and late retirement (17). However, few of the included studies actually considered working beyond the normal pension age. By contrast, the present study following individuals until at least one year beyond the SPA found that time pressure was a barrier. Altogether, this suggests that workplaces should strive to organize the work in a way that allows for reasonable workloads. The lack of prospects for job advancement was also a significant barrier to working beyond the pension age. This finding suggests that older workers are less likely to continue working if they perceive limited opportunities for career growth or feel stuck in their current position.

The combined overall score of the nine work factors showed the strongest association with working beyond the SPA, highlighting the importance of considering the work environment as a whole. This finding aligns with previous research, showing that the combinations of several psychosocial factors (18) as well as the combination of several ergonomic factors (19) are important in the prevention of long-term sickness absence. As long-term sickness absence is a strong prognostic factor for involuntary early retirement (20), addressing multiple aspects of the work environment simultaneously may be crucial for extending working lives. Thus, workplaces should focus on creating a supportive and engaging work environment that addresses multiple work factors, such as skills development, recognition, workload management, and career advancement opportunities.

In addition to the work factors, the secondary analyses of the present study showed that other factors also influenced working beyond the SPA. Although many of these cannot be directly altered through interventions, they provide valuable insights into the complex interplay of individual characteristics and societal factors that shape retirement decisions. Regarding marital status, those being divorced or separated were more inclined to work beyond pension age. This finding may be explained by increased expenses or a greater need for social interactions among people living alone (21). Thus, continued employment may provide the needed financial and social stability. Sex did not influence working beyond the SPA in the present study. By contrast, a common finding from previous studies is that women retire before men (22, 23). As a likely explanation for the contrast between the present and previous studies, we used the sex-, country- and year-specific SPA as reference for working beyond SPA. By contrast, previous research has often studied the risk of leaving the labor market at a fixed age, even though the SPA is lower for women than men in many European countries (2, 9, 10). Thus, the discrepancy between the present and previous studies may partly be due to different definitions of retirement.

Some of the present findings were not surprising. People who had never smoked were more inclined to work beyond the SPA. Although we controlled the analyses for self-rated health, non-smokers generally have better health (24), which influence the capability of working until a high age (25). Similarly, those with higher levels of education were more likely to continue working beyond the SPA, which may be explained by multiple factors such as health, work demands and work content. Surprisingly though, we did not find that self-rated health was associated with working beyond the SPA. By contrast, a Dutch study showed that health was important for working beyond retirement (5, 6). The findings of the present study may reflect a certain “healthy worker effect”, ie, participants were on average 57 years at baseline. Thus, many of those with poor health may already have left the labor market. Therefore, results regarding health might have been different if we had been able to include much younger people at baseline and following them for decades.

Interestingly, there were substantial differences in working beyond the SPA across European regions. Eastern, Southern, and Western Europeans were less inclined to continue working past the SPA compared to Northern Europeans. Various factors may influence retirement decisions across the European regions, such as labor market conditions, possibilities for early retirement, societal attitudes, cultural norms, and policy measures. Reforms in public pension schemes, such as raising the statutory retirement age, eliminating early retirement options, and providing incentives for working longer, influence the decision to continue working (26). Despite the regional differences observed in the present study, previous data from SHARE indicates an increase in planned retirement ages across European countries, albeit with variations among countries (27).

### Limitations and strengths

The strengths of this study include the large sample size, prospective cohort design, and inclusion of multiple European countries, which enhance the generalizability of the findings. The use of country-specific statutory retirement ages is another strength, although it may also be considered a limitation as we could not take into account the various early and flexible retirement pension schemes in the different countries. On the other hand, in the present study we were interested in the topic of working beyond the SPA and not early retirement. Another strength is that two sensitivity analyses confirmed the main findings, ie, the estimates for the work factors remained robust. The study also has some clear limitations. There is a chance of selection bias given the relatively low number of individuals fulfilling the inclusion criteria and the high drop-out or individuals with missing work status at follow-up. Additionally, the inability to include younger participants at baseline limits the understanding of factors influencing working beyond the pension age over the life course. Future research should aim to address these limitations by conducting longitudinal studies that follow participants from a younger age and exploring the effectiveness of workplace interventions designed to address the identified barriers and facilitators. Another limitation is that SHARE do not contain typical scales of work factors, such as those used in the Danish Psychosocial Questionnaire (28), but only single-item questions. This may lead to certain misclassification bias and reduced measurement accuracy. It is also a common challenge in longitudinal studies that covariates (eg, smoking status, marital status, self-rated health and BMI) may change over time. There is also risk of certain misclassification bias of the outcome due to the variable time between SPA and follow-up. Thus, some individuals may have worked beyond SPA for at least one year but not be working at the time of follow-up, and therefore not being classified as working beyond SPA despite meeting the criteria. We therefore adjusted the analyses for the individual follow-up time beyond SPA. Finally, the dataset used for the present analyses was not sufficiently large to allow for country-specific stratification. Future studies should conduct country-specific analyses with adequately sized samples to investigate whether the observed findings vary across different national contexts.

### Concluding remarks

This prospective cohort study across 26 European countries identified several modifiable work factors that influence working beyond the statutory pension age. Opportunities for skill development and recognition at work were facilitators, while time pressure and poor prospects for job advancement were barriers. These findings underscore the importance of creating supportive and engaging work environments addressing multiple factors to promote longer and healthier working lives.

## Supplementary material

Supplementary material

## Data Availability

SHARE data is publicly and freely available at share-eric.eu/data.
