# Supplementary material for: Facilitators and barriers for working beyond statutory pension age: A prospective cohort study across 26 European countries
Source: Scand J Work Environ Health. 2024 Nov 28;50(8):622–30. doi: 10.5271/sjweh.4189 (PMC11618848; doi:10.5271/sjweh.4189)

Facilitators and barriers for working beyond statutory pension age: A prospective cohort study across 26 European countries[1]

by Lars Louis Andersen, PhD,[2] Joaquín Calatayud, PhD, Rodrigo Núñez-Cortés, PhD, Ana Polo-López, MSc, Rubén López-Bueno, PhD

1. Supplementary material
2. Correspondence to: Lars Louis Andersen, National Research Centre for the Working Environment, Lersø Parkalle 105, DK-2100 Copenhagen, Denmark. [E-mail: lla@nfa.dk]

Appendix Figure 1. Development of statutory pension age (SPA) for men across 26 European countries from 2004 to 2022.

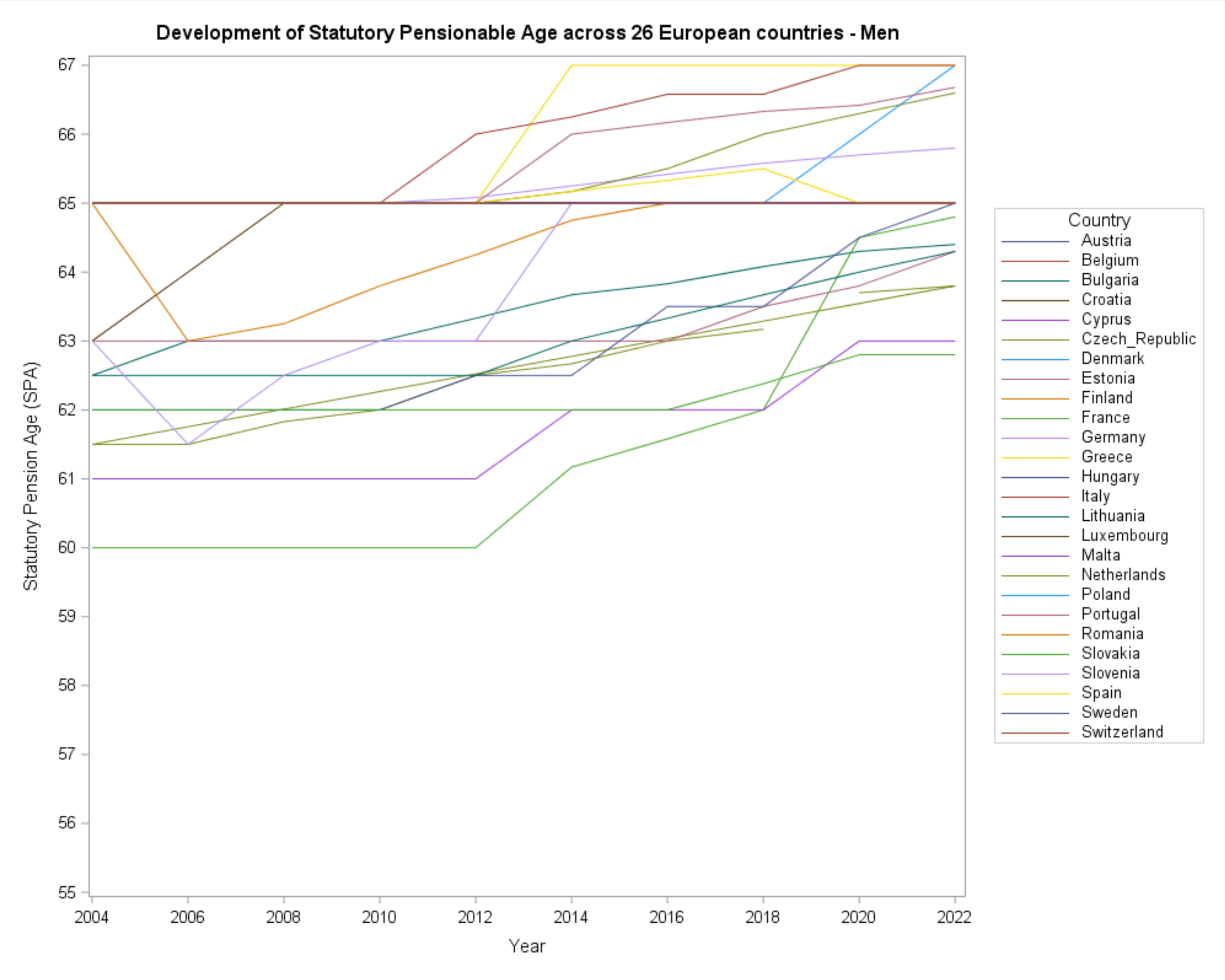

Appendix Figure 2. Development of statutory pension age (SPA) for women across 26 European countries from 2004 to 2022.

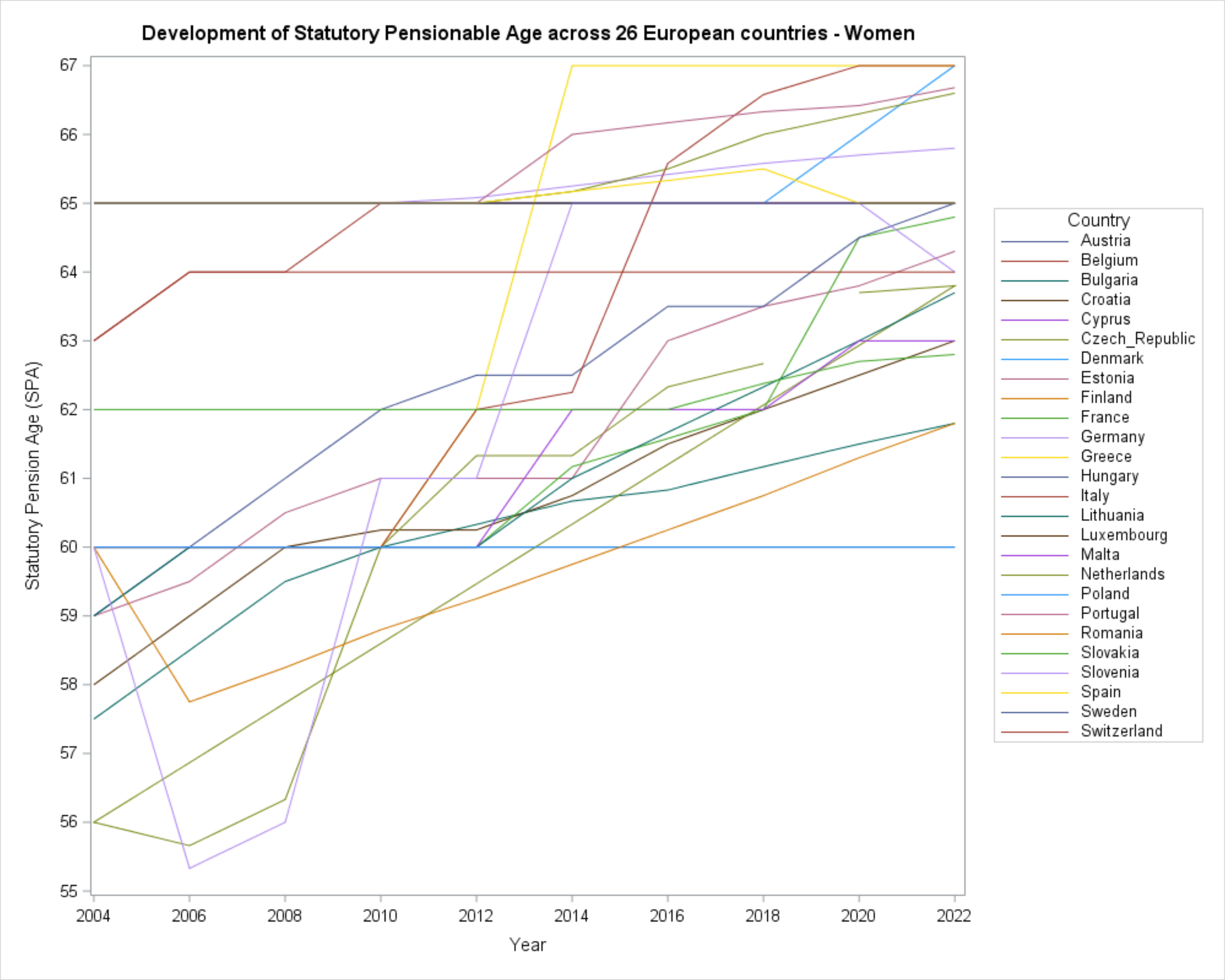

Appendix Figure 3. Development of statutory pension age (SPA) for men across the four European regions from 2004 to 2022.

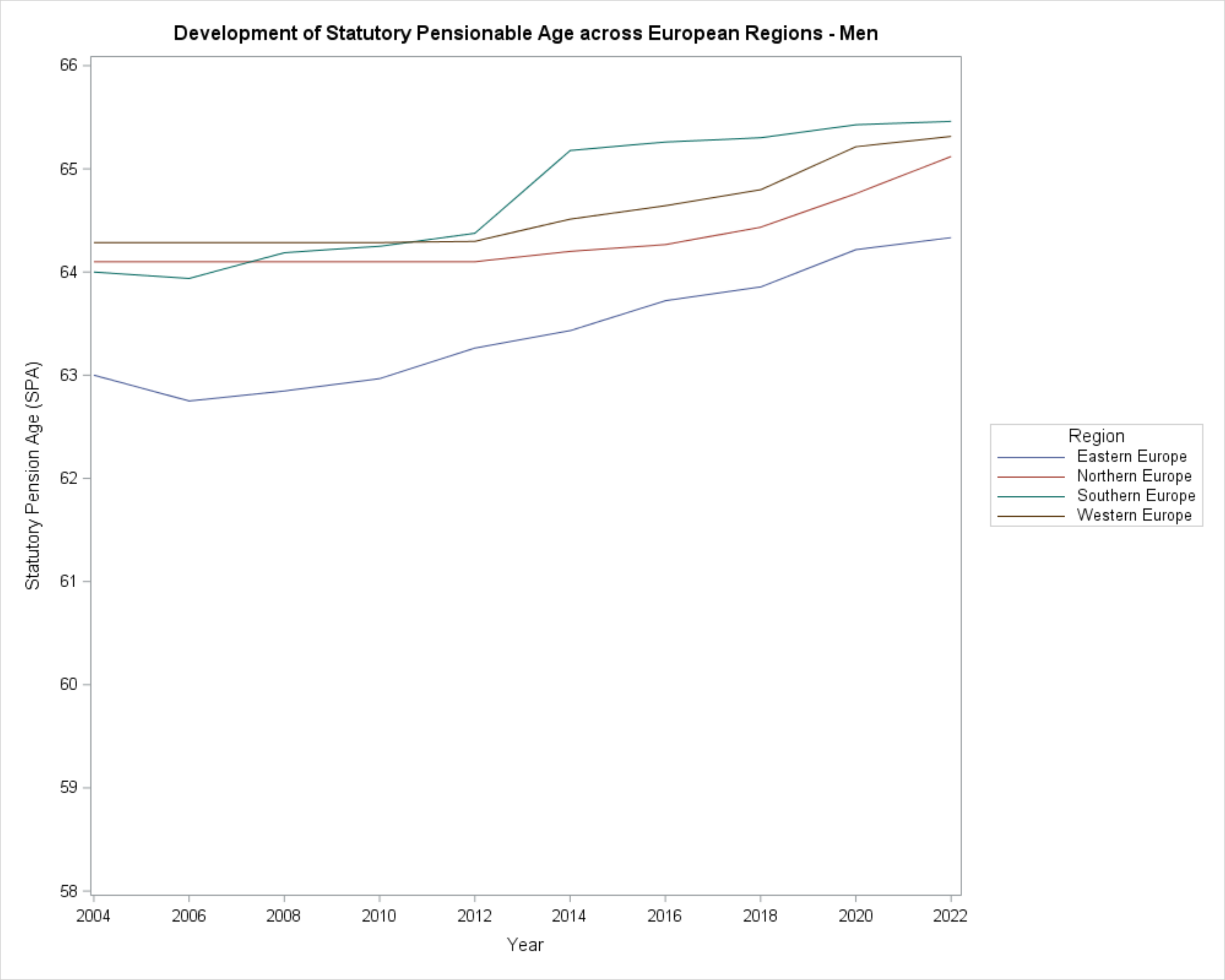

Appendix Figure 4. Development of statutory pension age (SPA) for women across the four European regions from 2004 to 2022.

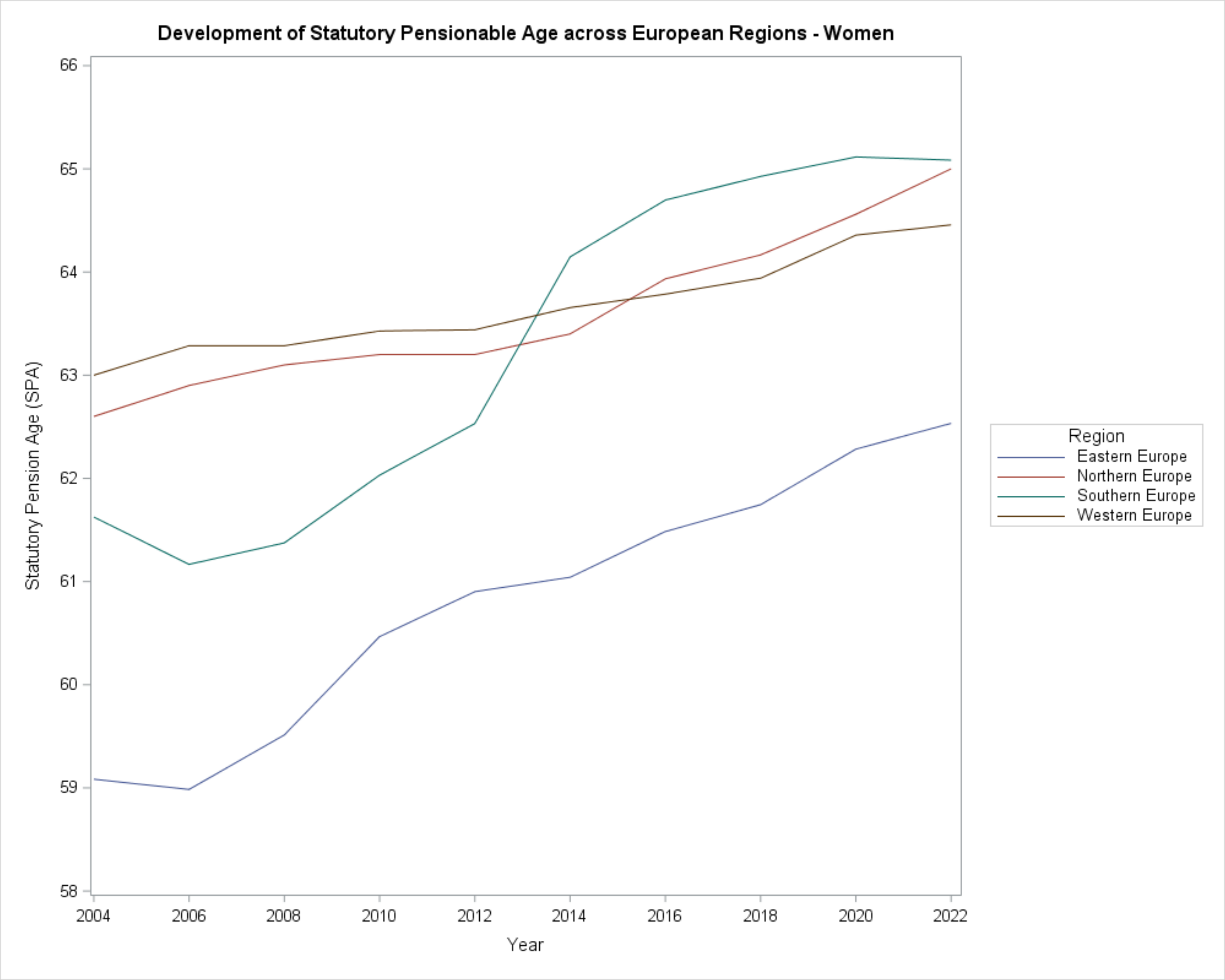

Supplement: Supplementary material [file SJWEH-50-622-S001.pdf]
